# Advancing Cardiac Surgery Case Planning and Case Review Conferences Using Virtual Reality in Medical Libraries: Evaluation of the Usability of Two Virtual Reality Apps

**DOI:** 10.2196/12008

**Published:** 2019-01-16

**Authors:** Sandeep Napa, Michael Moore, Tania Bardyn

**Affiliations:** 1 Department of Biomedical Informatics and Medical Education School of Medicine University of Washington Seattle, WA United States; 2 Health Sciences Library University of Washington Seattle, WA United States

**Keywords:** virtual reality, cardiac surgery, usability study, system usability score, NASA-Task Load Index, medical libraries, case planning, presurgical planning

## Abstract

**Background:**

Care providers and surgeons prepare for cardiac surgery using case conferences to review, discuss, and run through the surgical procedure. Surgeons visualize a patient’s anatomy to decide the right surgical approach using magnetic resonance imaging and echocardiograms in a presurgical case planning session. Previous studies have shown that surgical errors can be reduced through the effective use of immersive virtual reality (VR) to visualize patient anatomy. However, inconsistent user interfaces, delegation of view control, and insufficient depth information cause user disorientation and interaction difficulties in using VR apps for case planning.

**Objective:**

The objective of the study was to evaluate and compare the usability of 2 commercially available VR apps—Bosc (Pyrus Medical systems) and Medical Holodeck (Nooon Web & IT GmbH)—using the Vive VR headset (HTC Corporation) to evaluate ease of use, physician attitudes toward VR technology, and viability for presurgical case planning. The role of medical libraries in advancing case planning is also explored.

**Methods:**

After screening a convenience sample of surgeons, fellows, and residents, ethnographic interviews were conducted to understand physician attitudes and experience with VR. Gaps in current case planning methods were also examined. We ran a usability study, employing a concurrent think-aloud protocol. To evaluate user satisfaction, we used the system usability scale (SUS) and the National Aeronautics and Space Administration-Task Load Index (NASA-TLX). A poststudy questionnaire was used to evaluate the VR experience and explore the role of medical libraries in advancing presurgical case planning. Semistructured interview data were analyzed using content analysis with feedback categorization.

**Results:**

Participants were residents, fellows, and surgeons from the University of Washington with a mean age of 41.5 (SD 11.67) years. A total of 8 surgeons participated in the usability study, 3 of whom had prior exposure to VR. Users found Medical Holodeck easier to use than Bosc. Mean adjusted NASA-TLX score for Medical Holodeck was 62.71 (SD 18.25) versus Bosc’s 40.87 (SD 13.90). Neither app passed the mean SUS score of 68 for an app to be considered usable, though Medical Holodeck (66.25 [SD 12.87]) scored a higher mean SUS than Bosc (37.19 [SD 22.41]). One user rated the Bosc usable, whereas 3 users rated Medical Holodeck usable.

**Conclusions:**

Interviews highlighted the importance of precise anatomical conceptualization in presurgical case planning and teaching, identifying it as the top reason for modifying a surgical procedure. The importance of standardized user interaction features such as labeling is justified. The study also sheds light on the new roles medical librarians can play in curating VR content and promoting interdisciplinary collaboration.

## Introduction

### Background

Cardiac surgery is quite often a complex task. Valvular heart surgery (eg, mitral valve repair) and surgical management of adult congenital heart disease require detailed knowledge of patient-specific pathological and anatomical characteristics of the heart and great vessels to ensure patient safety and optimal surgical outcomes [1,2]. Three-dimensional anatomical reconstructions using two-dimensional data from radiographs, computerized tomography (CT) scans, or ultrasounds help surgeons previsualize a surgical intervention to define the surgical approach and navigation in the context of cardiothoracic surgery [3,4]. This is often accomplished with a headset, creating an immersive experience [5]. The use of virtual reality (VR) for clinical apps started in the early 1990s and has become more widespread with the availability of inexpensive computing power.

### Significance

Surgical errors can be reduced through the effective use of VR [6]. The ability to properly visualize complex spatial anatomy can potentially reduce operating room time and ensure better surgical outcomes. Planning the placement of surgical cannulae, incision length and position, placement of baffle, sizing the conduit, placement of a surgical patch, and choosing between a minimally invasive procedure versus an open procedure are all patient specific. Interactive VR visualizations of patient anatomy can benefit case planning and better inform patients to alleviate anxiety and provide consent for the procedure. The same VR model can also be used to train fellows, residents, and medical students [4,5,7,8]. Previously published literature has shown that trainees using VR simulators complete their surgical curriculum faster [4]. High-fidelity three-dimensional models are generally available for interactive visualization. However, there is a paucity of formal usability research on VR apps themselves for case planning purposes.

### Study Goals

We designed and implemented a study plan to compare VR software for use in presurgical case planning with cardiovascular surgeons. First, we identified gaps in current case planning approaches for elective cardiac procedures. Second, we evaluated the usability and utility of 2 commercially available VR interfaces for surgical case planning purposes. Finally, we explored how medical librarians and informaticians can play a role in graduate medical education and clinical information management.

## Methods

### Study Design and Setting

Through a mixed-methods qualitative study, we evaluated 2 commercially available VR apps: Bosc version 4.5 (Pyrus Medical systems) and Medical Holodeck version 2.0 (Nooon Web & IT GmbH). Semistructured individual ethnographic interviews were conducted before and after the usability study to understand the context of our findings. We employed a concurrent think-aloud protocol for the usability study, conducted in the University of Washington (UW) Health Sciences Library [9]. Surgeons, fellows, and residents were invited to participate in our study. The UW institutional review board approved the study.

Our usability study was an effort to help medical libraries to create their own VR and augmented reality services to help clinicians plan surgical cases and train residents and fellows. We collaborated closely with faculty and researchers affiliated with the UW Center for Cardiovascular Innovation (CCVI) laboratory. Through them, we were able to generate sufficient interest in the cardiology and cardiothoracic surgery departments at our institution, UW Medicine. The feedback we received from designing and implementing an innovation lab in a library space for VR app testing informed our usability study.

The VR usability testing was conducted in the UW Health Sciences Library’s Translational Research and Information Lab (TRAIL). The room and testing set up included the Textbox 1.

### Participant Selection

Recruiting volunteers to test VR was accomplished by posting an email to the resident listserv and departmental listserv at UW Medicine. Volunteers were invited to participate via email in a 1-hour usability session in TRAIL. Our recruitment window was open for 1.5 months (May to mid-June 2018), with 8 physicians taking part in the study. Our exclusion criteria included a history of epilepsy or motion sickness exacerbated by exposure to virtual environments. However, none of our respondents fit the exclusion criteria.

**The room and testing set up.**

- HTC Vive virtual reality (VR) headset and controllers
- VR-capable gaming laptop (MSI GT73VR Titan Pro laptop, Intel i7, 16 GB RAM, 1 TB hard disc drive, 128 GB solid-state drive, NVIDIA GeForce GTX 1080)
- 14 ft × 12 ft dedicated standing VR play area
- Six-screen ultra-high-definition data wall
- High-speed Wi-Fi connection to stream content, as required

### Study Protocol

After taking informed consent, the study team invited the participant to fill out a prestudy questionnaire in TRAIL to build out a user profile about activities related to case planning and issues faced during case presentations. The prestudy questionnaire included questions such as:

- Have you played computer games or participated in virtual simulations before? If yes, how many times in the past 2 years?
- Have you modified your surgical plan after you started operating on a patient recently? If yes, why?
- Could this information have surfaced during a case presentation?
- What do you want Virtual Reality to do for you?
- What are some other gaps you see during case presentations?

**Figure 1:**
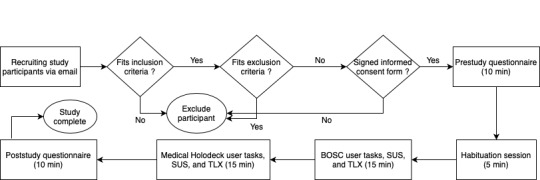
Study protocol. SUS: system usability scale; TLX: Task Load Index.

A habituation session (5 min) was conducted to familiarize the user with the VR interface around how to use the trackpad, navigate the play area, and ask for help if necessary. The goal of habituation was not to test the discoverability of a feature. It was to see how users combine basic interactions to achieve the endpoint of a scenario. A medical librarian observed the session to understand how to incorporate information into VR experiences in the future. Once the user was habituated, a 30-min usability study was conducted, with the time evenly split between first Bosc and then Medical Holodeck. A visual representation of our study protocol is provided in Figure 1.

User scenarios were sketched out keeping in mind all user tasks that need to be performed to complete the scenario. We had the following scenario for Bosc:

Scenario: You were given the CT scan of this patient with a lung tumor. Replicate this image and annotate the mass saying “Tumor.”

Hint: The patient image is on the last one on the lower right. Notice the density and opacity settings.

**Figure 2:**
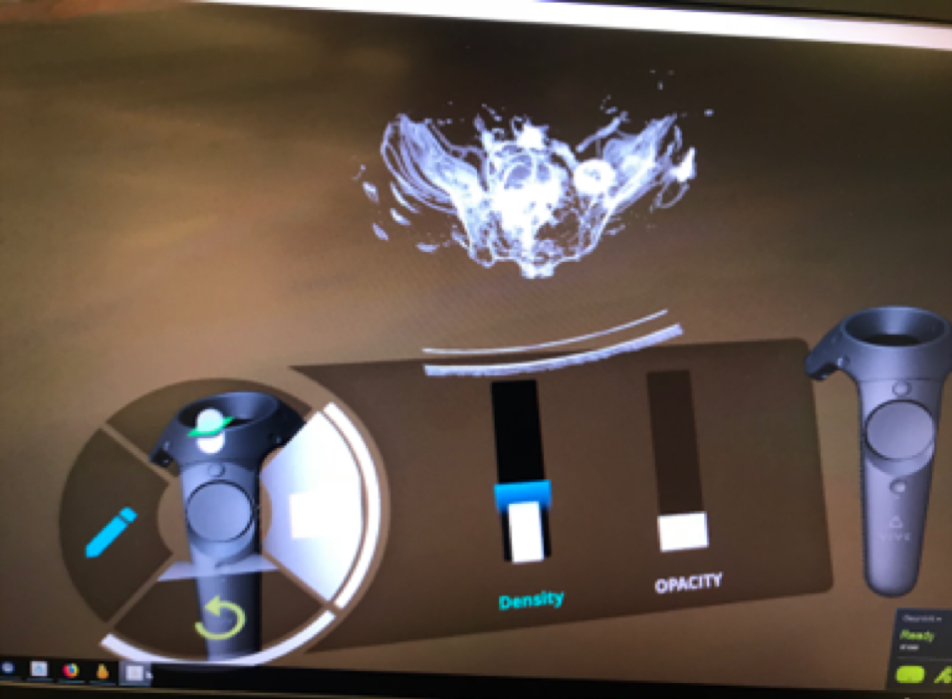
User interface of the BOSC.

For a screenshot of the Bosc interface please refer to Figure 2.

The tasks to accomplish the endpoint of this scenario were selecting an image, selecting the square tool, moving the sliders into optimal position, and selecting the annotation tool and marking the tumor.

The following was the scenario with 2 different endpoints for Medical Holodeck:

Endpoint 1: Two cut planes

Scenario: You are trying to visualize different structures in the chest cavity using the volumetric images provided to you by the radiology department. Can you replicate the following images?

The tasks to accomplish Endpoint 1 of this scenario were selecting the heart model, rotating the heart model, finding and using 1 cut plane, removing cut plane and using 2 cut planes.

Endpoint 2: Visualizing structures

Hint: Use −400 to −600 on the outermost filter ring and turn the rest off.

The tasks to accomplish Endpoint 2 of this scenario were: selecting the lung model, turning on and off ring filters, and adjusting the resolution on the outermost ring filter and turning off other filters.

**Figure 3:**
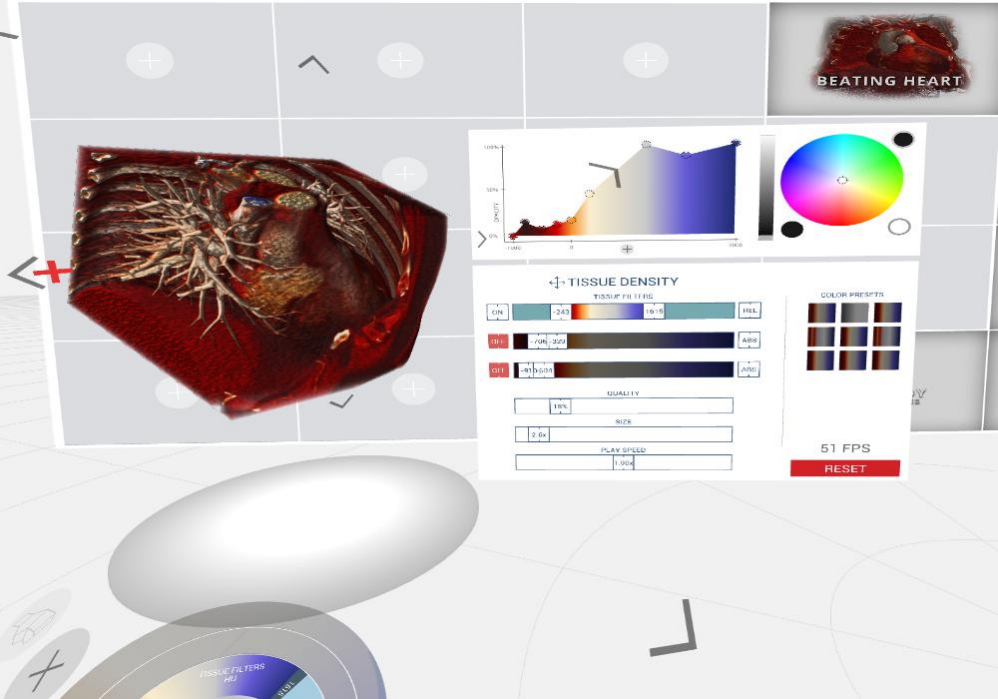
User interface of the Medical Holodeck.

For a screenshot of the Medical Holodeck interface please refer to Figure 3.

An observer noted verbal user feedback and task completion times. After each interface was tested, a questionnaire was administered to evaluate user satisfaction via 2 standardized tools: the system usability scale (SUS) and the National Aeronautics and Space Administration-Task Load Index (NASA-TLX). The questionnaires took approximately 10 min to complete. At the end of the study, users filled out a poststudy questionnaire, which included the following questions:

- Can VR make your case presentations easier? (Yes/No/Unsure) Why?
- What did you like about your experience?
- What did you think was missing?
- How would you prefer to use VR for case presentation? Single person mode (where you operate and present) or Presenter operator mode (where you present, and a colleague operates the VR)? Why?
- What else do you think VR can do for you?

The whole session lasted for 1 hour. Participants had the option to opt out of answering any question and the ability to opt out of testing at any time.

### Outcome Measures

We were interested in the usability and the utility of the VR apps and the role medical libraries could play to ease adoption of VR in clinical settings. Through prestudy questionnaires we identified gaps in current case planning approaches for elective cardiac procedures.

Through a poststudy questionnaire and a semistructured interview we explored the role of medical librarians and informaticians in graduate medical education and clinical information management.

### Analysis Approach

We presented participant characteristics and the 3 dimensions of usability (effectiveness measured by completion rate, efficiency measured by task completion time, and satisfaction measured by SUS and NASA-TLX). We also presented a qualitative analysis of responses to our prestudy and poststudy questionnaires, addressing the 3 aims of our study. Content analysis was performed on the ethnographic interviews. In addition, 2 of the investigators (SN and MM) reviewed notes and video recordings to identify key phrases. Both investigators performed this task independently and then met to agree upon the categories of feedback. Quotes were extracted to ensure accuracy. Task completion rate is defined as the proportion of users completing the task without assistance from the moderator. Task completion time is defined as the time it took in seconds for a certain task to be completed. Task completion parameters were defined, and the moderator confirmed user comprehension, before the participants started a task. Qualitative data were managed using Microsoft Excel (Microsoft Corporation). Quantitative data were managed using a Web-based app called Plotly (Plotly Technologies Inc, Montreal).

## Results

### Study Participant Characteristics

We reached out to approximately 60 faculty, fellows, and residents of whom 11 responded (11/60, 18%). We were able to schedule 8 users in our recruitment window. There were 63% (5/8) male participants and 38% (3/8) female participants. Our user sample had 6 faculty, 1 resident, and 1 fellow. We had a varied range of ages (29-69 years) and clinical experience (3-25 years) in our user group. On an average, 5 cases were presented per week per user (Table 1).

**Table 1:** Characteristics of study participants (n=8).

Characteristics of participants (surgeons)		Statistics
**Physician training level, n (%)**		
	Resident postgraduate year 1-4	1 (13)
	Fellow	1 (13)
	Physician	6 (75)
**Gender, n (%)**		
	Female	3 (38)
	Male	5 (63)
**Age in years**		
	Mean age, (SD)	41.5 (11.67)
	Median age, (min-max)	39.5 (29-69)
**Clinical experience in years**		
	Mean clinical experience, (SD)	13 (9.82)
	Median clinical experience, (min-max)	11.5 (3-35)
VR^a^ technology comfort level or exposure (past experience with three-dimensional computer games or VR simulations), n (%)		4 (50)
Case conference presentation frequency		5 cases/week

^a^VR: virtual reality.

### Usability Test

**Figure 4:**
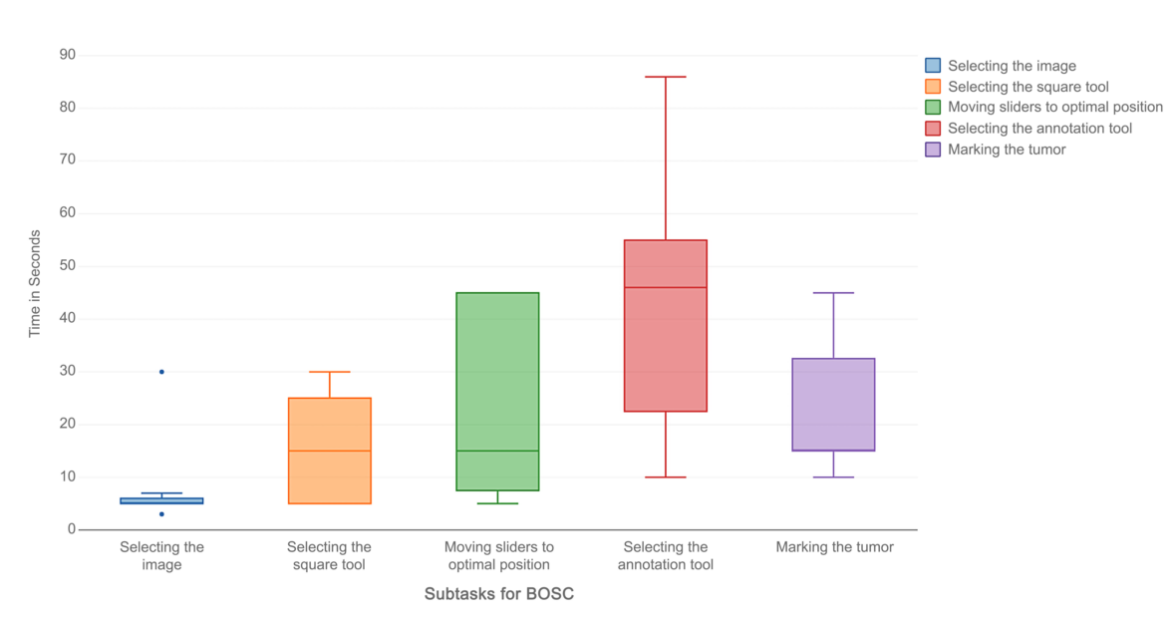
Task completion times for BOSC.

**Figure 5:**
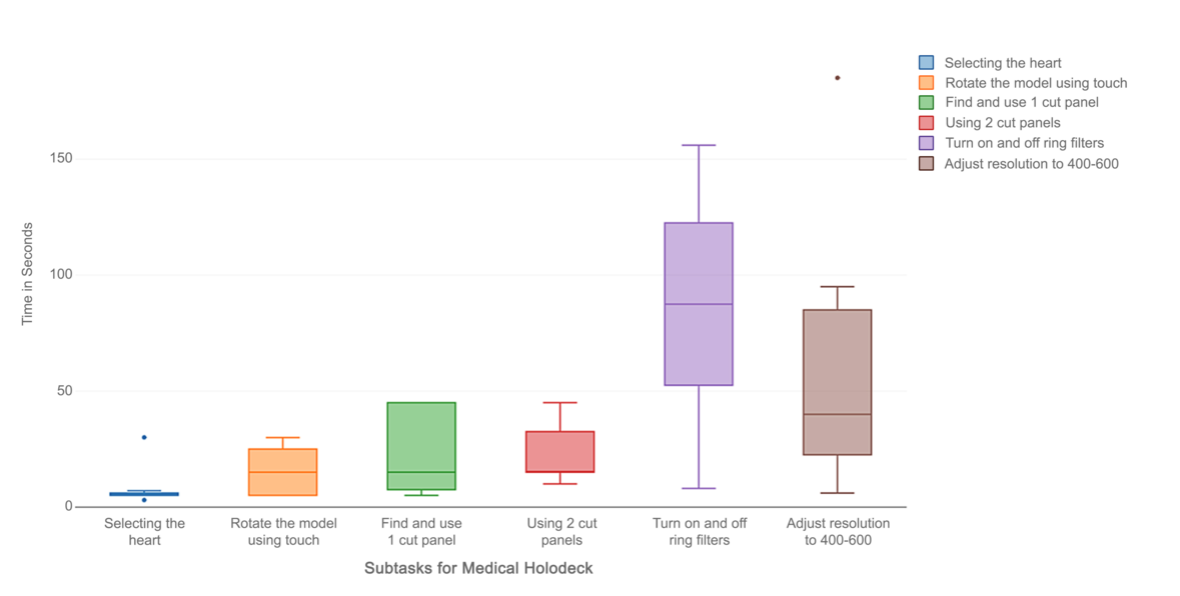
Task completion times for Medical Holodeck.

Effectiveness is a dimension of usability that can be measured using task completion rate, and efficiency is measured using task completion time. Certain subtasks such as selecting an image (a model), selecting a tool, marking a tumor, and using cut planes had 100% task completion rate and a short task completion time. Moving slider elements to an optimal position and selecting the annotation tool in Bosc had the worst task completion rate (0%) and the highest mean task completion time (154.57 seconds and 133.5 seconds, respectively). The same pattern was observed in Medical Holodeck. The subtasks with the worst completion times (25%) were specific to the app (eg, removal of cut planes and turning filters on and off) and had the longest mean completion times (42.88 seconds and 86.13 seconds, respectively). Another task that had a poor completion rate (50%) was the adjustment of filters to a certain window, which also had a long mean task completion time of 60.75 seconds (Table 2; Figures 4 and 5).

### User Satisfaction

We used the TLX to measure cognitive burden and the SUS to measure usability of each app. These are considered good measures of user satisfaction [10-12]. Subjective workload depended on the frustration the user faced with each app. In detail, there were 3 elements of the scale that contributed to most workload among users. “Frustration” was the most common (“How insecure, discouraged, irritated, stressed, and annoyed were you?”), followed by “Performance” (“How successful were you in accomplishing what you were asked to do?”), “Temporal demand” (“How hurried or rushed was the pace of the task?”), and “Mental demand” (“How mentally demanding was the task?”). No users found the apps physically demanding, as evident in the low weights it received (Table 3).

Bosc had a higher cognitive burden mean TLX score (62.71 vs 40.87) and a lower mean SUS score (37.19 vs 66.25). However, neither app passed the mean SUS score of 68 for an app to be usable [10]. Medical Holodeck was found usable by 3 users, whereas Bosc was rated usable by a single user (Table 4).

**Table 2:** Task completion times and task completion rate.

Task		Mean (SD), in seconds	Unassisted completion rate, %
**Bosc**			
	Selecting the image	4 (1.12)	100
	Selecting the square tool	6.36 (3.84)	100
	Moving sliders to optimal position	154.57 (46.89)^a^	0
	Selecting the annotation tool	133.5 (55.10)^a^	0
	Marking the tumor	17.5 (17.14)	88
**Medical Holodeck**			
	Selecting the heart	8.13 (8.33)	75
	Rotate the model using touch	15.83 (9.32)	96
	Find and use 1 cut plane	23.13 (17.31)	75
	Remove cut plane	42.88 (22.91)	25
	Using 2 cut planes	22.5 (11.73)	100
	Turn on and off ring filters	86.13 (45.74)	25
	Adjust resolution to 400-600 on the outermost ring filter and turn off other ring filters	60.75 (54.38)	50

^a^Completed with assistance.

**Table 3:** Weighted dimensions of the National Aeronautics and Space Administration-Task Load Index (NASA-TLX).

User	Mental demand	Physical demand	Temporal demand	Performance	Effort	Frustration
User 1	2	0	1	4	3	5
User 2	3	1	4	5	2	0
User 3	2	0	4	4	1	4
User 4	4	0	1	2	3	5
User 5	5	1	2	4	3	0
User 6	4	1	4	0	2	4
User 7	4	1	1	1	3	5
User 8	2	0	5	3	2	3
Mean	3.25	0.5	2.75	2.875	2.375	3.25

**Table 4:** Results from the system usability scale (SUS).

SUS^a^ (out of 100)	Bosc	Medical Holodeck
Score, mean (SD)	37.19 (22.41)	66.25 (12.87)
Users who rated the app usable (SUS >67), n (%)	1 (13)	3 (38)

^a^SUS: system usability scale.

### Usability Problem Breakdown

Sliders were considered well-known interface elements because all of our users use mobile devices and were familiar with the slider interface to change a setting. Using a cut plane or ring filter, for example, had no parallels in everyday user interfaces so we considered them less commonly known. Frequency represents the fraction of the number of users who faced a certain usability problem over all users (n=8) (Table 5).

### Ethnographic Interviews

The most commonly voiced issues in case presentation were inaccurate or unclear communication of patient anatomy (3/8, 38%), difficulties in teaching (2/8, 25%), and varying image interpretations (2/8, 25%) (Table 6). Intraoperative findings or anatomical considerations were the most common reason to modify surgical plans (4/8, 50%). Users were unclear about the perceived impact of surgical plan modification (3/8, 38%) and were not sure if the information that led to these modifications could have surfaced during case planning (7/8, 88%). The most commonly perceived gap in case presentation was communicating anatomical details (50%). The most desired benefits from implementing VR were improving imaging of complex cases (3/8, 38%), improving communication (2/8, 25%), and the ability to afford better planning (2/8, 25%) were both high on the list.

Our poststudy questionnaire explored the utility of VR and how librarians could play a role in curating and collaborating around VR. Users liked learning about VR (5/8, 63%) and knowing what is new out there (3/8, 38%). Some users found it difficult to understand the clinical context of VR apps (3/8, 38%) and whether they gave us useful information (2/8, 25%). Most users wanted a single operator-presenter system (4/8, 50%) instead of a dual separate operator and presenter setup. There was overwhelming emphasis on using VR for training (7/8, 88%) and patient education (4/8, 25%). The role of librarians, as our user group suggested, should be around providing a teaching resource via a repository of VR images collected by clinicians (3/8, 38%), providing space, apps, and equipment (3/8, 38%). However, most users were unsure (4/8, 25%) about the role librarians can play in clinical information management.

**Table 5:** Analysis of usability problems.

Usability problem type		Description	Frequency	Severity^a^
**Bosc**				
	Using a well-known interface element in a virtual environment	Selecting and moving sliders to desired position	7/8	Medium
	Using a well-known interface element in a virtual environment	Using the annotation tool	7/8	Medium
	Software errors	Delayed slider movements	1/8	Low
	Slider sensitivity	Higher sensitivity requires users to be cautious	3/8	Medium
**Medical Holodeck**				
	Using a well-known interface element in a virtual environment	Rotating the heart model	3/8	Low
	Using a less commonly known interface element	Creating and removing cut planes	6/8	Medium
	Using a less commonly known interface element	Turning ring filters on and off	6/8	Medium
	Using a less commonly known interface element	Adjusting ring filter resolution to specification	6/8	Medium

^a^Severity scale: low: task was delayed; workaround unnecessary; medium: task was delayed, workaround was necessary, or moderator helped the user; high: task was delayed or left incomplete, user couldn’t complete the task even with moderator’s assistance.

**Table 6:** Results of the ethnographic interviews (n=8).

Characteristic		Statistics, n (%)
**Issues in case presentation**		
	Inaccurate or unclear communication of patient anatomy	3 (38)
	Teaching difficulties for new learners	2 (25)
	Varying image interpretations	2 (25)
	Conveying the acuity of the clinical situation	1 (13)
	Ease of bringing up relevant imaging in clinic or operating room	1 (13)
	Not knowing what anatomy will look like in real time	1 (13)
	Special training and software requirement for assessing MRI^a^	1 (13)
	Limited applicability of some technologies	1 (13)
**Reason to modify surgical plans**		
	Anatomy or intraoperative findings	4 (50)
	Imaging inputs or new information from old surgical records	1 (13)
	Need to be innovative	1 (13)
**Perceived impact of surgical plan modification**		
	Unclear	3 (38)
	Increased operating room time	3 (38)
	Greater morbidity	1 (13)
	Anticipated improved outcome	1 (13)
**Could this information have surfaced during case planning?**		
	Maybe	7 (88)
	Yes	1 (13)
	No	0 (0)
**Gaps during case presentation**		
	Communicating anatomical details	4 (50)
	Case presenters unaware of priorities	1 (13)
	Lack of retrievable mental imagery	1 (13)
	Imaging limitations	1 (13)
	Equipment readiness and reliability	1 (13)
	Lack of clear problem statement and next steps	1 (13)
**Potential apps for VR^b^**		
	Improve imaging of complex cases	3 (38)
	Improve communication	2 (25)
	Better planning	2 (25)
	Dynamic and accurate measurements of anatomy	1 (13)
	Display anatomy of complex cardiac repairs	1 (13)
	Educate patients on complex cases	1 (13)
**Things liked about the VR experience**		
	Learning about new technology	5 (63)
	Knowing what is new out there	3 (38)
	Interesting interface	1 (13)
	Interesting anatomical models	1 (13)
	Clear instructions and specific tasks	1 (13)
	Interactive learning as you go	1 (13)
	Relaxed atmosphere	1 (13)
**Things missing in the VR experience**		
	Clinical context or applicability to respondent’s scope of practice	3 (38)
	Unsure if investigators were provided with useful information	2 (25)
	Benefit of VR over current systems	2 (25)
	Lack of understanding of controller setup before starting task	1 (13)
	Nothing	1 (13)
**Preferences for VR interface control**		
	Single person mode	4 (50)
	Both	2 (25)
	Only as an adjunct	1 (13)
	No answer	1 (13)
**Alternative apps of VR**		
	Trainee education	7 (88)
	Patient education	2 (25)
	Plan for appropriate devices necessary for treatment	1 (13)
	Warm up or practice	1 (13)
	Team communications	1 (13)
**Role of librarians in graduate medical education**		
	Teaching resource via repository of VR images collected	3 (38)
	Provide space, apps, and equipment	3 (38)
	Serve as part of the team	1 (13)
	Inform and educate the community	1 (13)
	Train on VR environment	1 (13)
	Invest in VR	1 (13)
**Role of library in graduate medical education**		
	Unsure	3 (38)
	Increase access to case materials for presentations	2 (25)
	Find more apps	1 (13)
	Provide strategies for research into clinical topics	1 (13)

^a^MRI: magnetic resonance imaging.

^b^VR: virtual reality.

## Discussion

### Principal Findings

**Figure 6:**
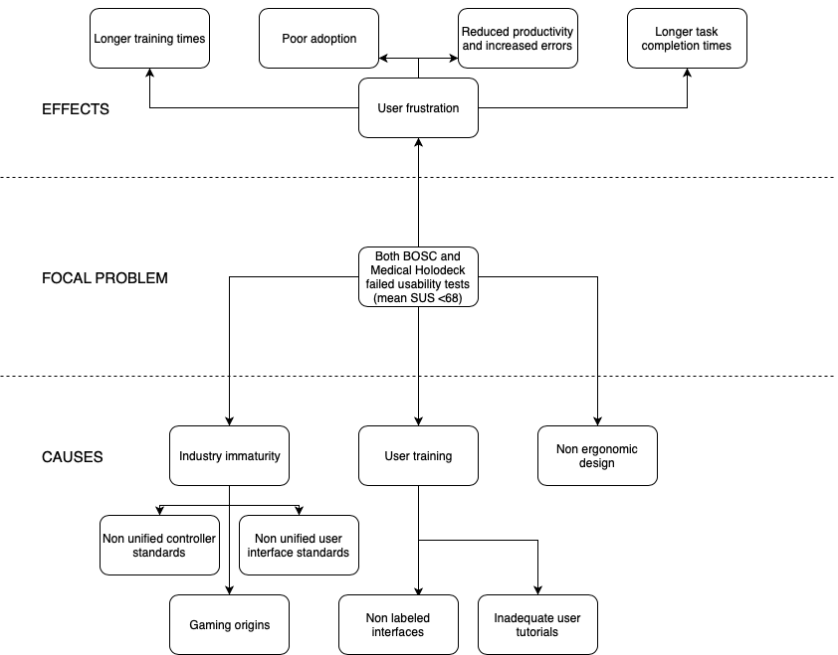
Problem tree analysis. SUS: system usability scale.

In a usability study with 8 surgeons, resident physicians, and fellows at the UW, each user spent 60 min testing 2 VR apps—Bosc and Medical Holodeck. Users reported a general sense of frustration using the apps, but were appreciative of the role VR could play in case planning. Subtasks such as selecting a tool, marking a tumor, and using cut planes had high task completion rates, short task completion times, and less variation among users. This is likely because of these interactions being borrowed from daily life apps. When users were unaware of how to use a feature or when the interaction was app specific with no parallels to real-life apps, we observed poor task completion rates, higher inter-user variations, and long task completion times (Figure 6). Some users found the gesture-based controls confusing. User comments that demonstrate the frustration with these controls are quoted verbatim in Textbox 2.

**User comments that demonstrate the frustration with the controls.**

- “Draw. That should be obvious. *tries to draw* But it’s not actually doing what it says.”
- “The direction in scrolling doesn’t always match where the slider is going.”
- “I’m trying to figure out how to move between the bars. I haven’t figured out how you control it.”
- “Did you guys do that or did I? I didn’t do anything, and it moved. Is somebody else doing something?”
- “It’s moving both. I can’t control one without the other.”

The same pattern was observed with Medical Holodeck. However, unlike Bosc, the model selection interaction was not understood by all users. The subtasks with the worst completion times were specific to the app with no parallels to interactions users have in daily life, such as removal of cut planes and turning on and off the filters. These also had the longest completion times. Frustrations with the app are quoted verbatim in Textbox 3.

The app also required the user to hold his or her hands above waist level to continue visualizing the image, frustrating 1 user who said:

I can’t really drop my hands to my sides. That would be nice to be able to stand here looking at model [without having to hold up my hands].

Sensitivity of the controls and a slight lag in the user interaction was an issue identified by multiple users as shown in Textbox 4.

The user comments for Medical Holodeck were similar and are provided in Textbox 5.

The apps were not considered physically demanding by any users, as evident in the low weights it received.

### User Satisfaction

In general, users found Medical Holodeck easier to use (Textbox 6).

Users appreciated Bosc as well, but commented on its limitations (Textbox 7).

**Frustrations with the app.**

- “It’s hard to tell in the visualization where I’m clicking.”
- “If I click it just creates more planes.”
- “It’s very clunky.”

**Sensitivity of the controls and a slight lag in the user interaction.**

- “You just touch it and it switches [from opacity to density or vice versa].”
- “There’s a lag.”
- “I don’t understand why it’s moving at this point.”
- “Maybe I just have to push longer harder? I feel like I should just have to push on the trackpad, but it isn’t working.”
- “Very confusing. It was tough to figure out what the buttons did. It seemed like I could never figure out what I was doing while it was happening.”
- “That’s less than ideal.”

**User comments for Medical Holodeck.**

- “Now I’m getting a little frustrated.”
- “I have the panel but I’m not sure how to change it.”
- “This is where I would expect the function on the left to stay lit up.”

**User satisfaction comments on Medical Holodeck.**

- “It’s good that it has labels, even if they don’t do what they say.”
- “I like the second app [Medical Holodeck]. I like the labeling that shows you what does what.”
- “It was easier to figure out what to do. The only thing was the laser; I wasn’t sure how far you have to be [to have it ‘catch’].”
- “It seemed crisper and a better viewing experience.”
- “It seemed more straightforward. It was clearer in terms of what each button does. It seemed more responsive.”
- “You didn’t have the sense that the pointer had as much power until [the moderator] told me it was what you had to use. Once you understand that it is easy to use.”

**User satisfaction comments on Bosc.**

- “I thought the app was pretty good, I just thought the scroll pad was awkward.”
- “It would be good if there were labels to say what things did.”

**Overall user impressions: positives.**

- “Once I got a sense of what you wanted me to do, and you’re not used to toys, it’s a left-brain, right-brain thing where you’re trying to do two things at once.”
- “I conveniently see instructions [labels], which is a step in the right direction.”

**Overall user impressions: negatives.**

- “The question is what can it do that I can’t do on my desktop?”
- “I’m not sold personally on this use of VR. That’s my own personal bias.”
- “It’s all about picking the right audience.”
- “From a practical point we’d really like to see where the blood vessels are.”

We observed that TLX and the SUS provide user satisfaction information in different dimensions, and that a mixture of metrics in the context of user interviews provides us better insights into user perception of these apps. For example, 1 user rated both apps similarly in the SUS. However, on comparing the frustration score in the TLX, we were able to uncover which specific interaction was the most challenging, which we could clarify in the poststudy interview. This approach would be of benefit to interaction designers for VR apps. One user said:

There’s no uniform approach to the button [in the HTC Vive]. Every time you go into a program you need to figure out what the buttons do.

Building a standardized user interface for VR requires time, just as the decade that smartphone interactions took to reach maturity.

### Questionnaire Analysis

Our prestudy questionnaire revealed interesting insights. The importance of precise anatomical visualization in presurgical planning and teaching is underscored by the fact that the most common issue in case presentation is not knowing how the patient anatomy will look like during the procedure. Similarly, intraoperative anatomical considerations were the most common reason to modify a surgical plan.

Our initial assumption was that users would prefer a 2-person mode of VR operation, where a surgeon presented the case and an operator (a fellow or resident) would navigate the VR system. However, most users wanted a single operator-presenter system. Considering the overwhelming emphasis on using VR for training and patient education and the relative immaturity of currently available VR apps, these are more viable apps than case planning for VR. As most users were unsure (3/8, 38%) about the role librarians can play in clinical information management, the librarians must make an active effort to communicate the value they bring to the table in curating clinical content and promoting interdisciplinary collaborations. One user suggested:

All surgeons require a retrievable system on which to think. Build a set of imagery they can recall. If you are training a team, you have to build that collection of images.

### Overall User Impressions

Although surgeons and resident physicians experienced individual challenges in using the 2 VR apps tested, the overall impression was positive (Textbox 8).

Working at an academic institution and teaching hospital, incorporating VR into ongoing and future teaching methods was of high interest to our faculty. Said 1 user:

We do not teach three-dimensional topics well. Almost all of our imagery is in 2 dimensions. Three dimensions make complexity better.

Not all users were excited about the prospects for VR, feeling that the apps were irrelevant or the immersiveness distracting (Textbox 9).

Our study results suggest that VR can be a useful adjunct in traditional presurgical planning methods, an observation also echoed by other studies in this domain which highlight the potential for group-based approaches, user-defined interactive views, and cost-effectiveness over 3D printing [13,14].

### Limitations

There are several limitations to the study. First, the study participants were self-selecting. A total of 37.5% of our participants had been exposed to immersive VR since they also worked with the CCVI. This may not be representative in other similar departments. Second, we only evaluated VR apps available to us. There are many other apps that are designed for specific purposes that we were unable to test. However, we have consolidated feedback to acknowledge user-friendly features of each app that serves as a benchmark to evaluate other such apps. Third, we had used existing VR models in these apps to avoid using actual patient data. Users, therefore, questioned the utility of these apps while identifying possible future research directions. Fourth, generating stereolithography models for VR apps requires high-resolution CT images, which we find difficult to acquire at our institution for most patients. This may impact future studies conducted at our institution. Finally, it is also possible that Medical Holodeck received higher usability ratings because it was the second app users tried. Multiple users indicated that they struggled or were frustrated earlier on in the testing but found it easier as they grew more accustomed and experienced to VR and the controllers, which coincides with their testing in Medical Holodeck. To preserve uniformity, however, we did not randomize which app the user tried first. In addition, we did not have enough users to draw statistically significant conclusions, even if we had randomized the order.

### Conclusions

We evaluated the usability and utility of 2 commercially available VR apps (Bosc and Medical Holodeck) for cardiothoracic case planning. We found that, on an average, neither app passes the minimum mean usability score of 68 on the SUS. Although users found Medical Holodeck less cognitively demanding (mean TLX score of 40.87 vs 62.71), more work is needed to make both apps usable. We also identified ways to make VR apps more useful in the clinical setting and for teaching. As we explore new apps, the role of medical librarians in curating VR content and promoting collaboration is evolving. Our hope is that medical libraries around the world benefit from our work and develop VR studios of their own for clinical apps.

## Abbreviations

CCVI: Center for Cardiovascular Innovation
CT: computerized tomography
NASA-TLX: National Aeronautics and Space Administration-Task Load Index
NIH: National Institutes of Health
SUS: system usability scale
TRAIL: Translational Research and Information Lab
UW: University of Washington
VR: virtual reality
